# ‘Perhaps a Bit Different to What We Did Twenty Years Ago’: Senior Teachers’ Perceptions of Outdoor Adventure within Primary Education in England

**DOI:** 10.3390/sports7040092

**Published:** 2019-04-19

**Authors:** Chris Webber, Ashley Hardwell

**Affiliations:** School of Sport, Leeds Beckett University, Leeds LS6 3QS, UK

**Keywords:** physical education, national curriculum

## Abstract

Outdoor and adventurous activities (OAA) are now a compulsory component of the primary education curriculum in England, with senior leadership teams exerting significant influence on its delivery in schools. This study considers senior teachers’ perceptions and value of the OAA strand of the Physical Education (PE) National Curriculum (NC) in primary education. Six senior teachers from across a large northern city took part in semi-structured interviews. Data was analysed using an interpretivist paradigm incorporating a multistage thematic coding process. Findings centred on the lack of guidance given by the NC within OAA and ensuing issues for experienced and less confident teachers of the subject. Different interpretations of OAA were prevalent from traditional skills-based activities to personal and social development through basic activities delivered outside the classroom. Finally, all senior staff highly regarded OAA and offered a strong rationale for its inclusion within curriculum time. The full potential of OAA as a cross-curricular approach to learning in primary education is not being realised and can be partially mitigated by more purposeful integration within teacher education programmes.

## 1. Introduction

Outdoor and adventurous activities (OAA) can elicit many positive health and well-being outcomes difficult to achieve via other means [1,2,3,4,5,6]. Consequently, OAA has been incorporated into British education for over a century [7,8,9,10,11,12,13,14]. Other benefits inextricably entwined with well-being typically include opportunities for holistic education [6], enhanced self-awareness and interpersonal skills [15,16,17,18,19,20,21] and cultivating life skills such as resilience, risk management, and independence [15,17,21,22,23,24,25]. OAA experiences create vivid long-term memories [15,23], through autonomy and exploration [1,17]. These activities often fall outside normal family experiences such as holidays or visiting friends and relatives [2,10,19,24] and can develop healthy lifelong habits [3,20,25,26] which positively influence academic attainment [15,19,20,27,28].

Since the inception of a National Curriculum (NC) in England in 1988, OAA has been formally, and somewhat contentiously, imbedded within Physical Education (PE) and seen as important in the development of transferable personal and social skills [10,14,16,19,28,29,30,31,32,33,34,35,36]. In 2014 the Conservative-led Government elevated the status of OAA from optional to mandatory from the age of seven (key stage two) onwards [29]. Primary education in England should be well placed to facilitate this recent curriculum shift. Schools now receive the School Sports Premium (SSPr), a significant ring-fenced fund (minimum of £16,000 per year, as of 2018) intended to improve mandatory PE and school sport provision, which includes OAA delivery [37,38,39].

Existing studies have shown senior leadership teams within schools to be pivotal in shaping school practices and cultures [19,25,31,40]. However, if knowledge and understanding is lacking and OAA not valued, this may affect its delivery [18,20]. This is often compounded by conflicting priorities placed on the school leadership team through Ofsted inspection, league tables and attainment in core subjects such as English and Mathematics [5,7,41,42,43,44]. Several studies have investigated OAA delivery within schools in England, but few have been conducted since the recent shift in NC policy and introduction of SSPr [25,27,28,40]. Empirically based knowledge is required to more fully understand primary education senior teachers’ perceptions and value of OAA because of the pivotal role they play in its promotion and delivery. This paper presents findings taken from a wider research project that investigated the complexity of delivering OAA within primary education in England. Specific objectives included how senior teachers interpreted the OAA strand of the NC, their understanding of OAA as a concept within an educational context, and the value placed on OAA within curriculum time. Six in-depth semi-structured interviews produced rich data presented under the findings of this paper.

## 2. Literature Review

### 2.1. Concepts of OAA

A myriad of overlapping concepts are used to refer to structured learning that occurs outside of classrooms [15,28]. Example terminology includes: Outdoor Learning, Outdoor Education, Adventure Education, Learning in Natural Environments and Outdoor Adventurous Activities [18,21,29,40,45,46]. Many of these have preconceptions. For example, teachers historically consider Outdoor Education (OE) to largely include skills-focused outdoor pursuits that incorporate elements of risk and challenge, often within a residential setting [14,25,28]. Fagerstam [15] supported this notion by stating OE is something typically provided by professional organisations at purpose built outdoor centres rather than schools. Similarly, Thorburn and Allison [35] suggested OAA is routinely perceived to encompass a week-long residential experience, which they asserted is a common misconception. OE has historically been preferred in Britain; however, Sutherland and Legge [14] suggested this term started to experience rivalry from Adventure Education around the 1990s. Despite international trends, the Government has adopted the term Outdoor and Adventurous Activities (OAA) within educational policy. Hence, for the purposes of this paper and to ensure consistency with existing legislation, OAA is used throughout to refer to activities typically occurring outdoors in natural environments which include components of challenge and adventure [14,29,47]. Primary school teachers often lack a clear and consistent conceptual understanding of both PE and OAA [28]. Coupled with the fact that generalist teachers gain scant guidance from the Physical Education National Curriculum (NCPE) and often do not receive much in the way of formal preparation regarding OAA, it is unsurprising many lack confidence in this area [14,21,24,40,48,49,50,51,52,53,54].

### 2.2. OAA in the National Curriculum

In the most recent NC (2014) the presence of OAA has been coupled with minimal specific guidance [29]. This has proven to be a strength in that broad links can be made between other curricular subjects, and a weakness, because teachers have the autonomy to deliver OAA how they see fit. Government has encouraged teachers to exercise professional judgement in translating and enacting the latest NC to fit within the socio-cultural context of their respective school [53]. This can cause difficulties for primary school teachers since many are non-subject specialists and lack PE and OAA related subject knowledge and pedagogical confidence [14,21,24,40,48,49,50,51,52,54]. Such findings are not confined to the UK. Recent research by Remington and Legge [43] and Dyment et al. [55] considered school teachers’ perceptions from an antipodean perspective, highlighting many professionals lacked clarity and understanding leading to OAA and its potential being undervalued. OAA documentation was open to interpretation and lacked clear working examples. They concluded curriculum change must be supported by effective teacher resources. Such findings have not been heeded in England. NC documentation generally, and PE and OAA specifically, has been produced to allow greater local autonomy, with this approach particularly problematic in primary education through lack of subject knowledge.

MacLean et al. [53] purported autonomy gaps can emerge between the intentions of policy makers and how delivery is successively translated into practice with unintended consequences possible as a result of ambiguity. Flintoff [56] referred to this concept as ‘slippage’ where practitioner delivery strays from the ideologies of policy makers. When considering marginalised areas of the curriculum such as OAA, this notion is concerning, since Flintoff explained the NCPE can be seen as a flexible document, open to debate and meaning different things to different schools. Empirical findings partially substantiated this concern, as teachers sought further clarification and support from senior management to affirm their interpretations of the policy were correct [53,56].

Influential work on policy by Penney and Evans [57,58] rejected the NC as a hierarchical document blindly adopted by others, instead arguing implementation is a process involving several stakeholders. This notion was substantiated by Curtner Smith’s early research [50] on teachers’ interpretations of the NCPE in England. Occupational Socialization was used as a theoretical lens to consider factors which influenced teachers ensuing practice. Findings highlighted teachers’ practice was markedly different to intended policy aims, with staff adapting, recreating, and modifying the NC to fit their own beliefs about teaching. Curtner-Smith [50] also found teachers with less experience were more likely to embrace new ideas, and those with more experience resist change by only modifying rhetoric rather than behaviour.

### 2.3. Valuing OAA in English Schools

Flintoff [56] explained policy shifts rely heavily on the enthusiasm of those involved in implementation, which in this context is teachers. Since Curtner-Smith [50] found teachers practice often reflects their values, it is important to consider this in relation to OAA, particularly given its shift to mandatory status [31]. Ofsted [49] reported curriculum ideas valued by senior teachers will often cascade down to inspire other colleagues. Pether [31] substantiated this notion stating teachers highlighted senior leadership teams as instrumental in successful initiation and development of an OAA culture within primary education. They saw head teachers as those responsible for providing the initial impetus behind a particular vision before sharing this responsibility amongst other staff. While Remmington and Legge [43] and Cosgriff [28] commented on this in the antipodean context, there is a dearth of empirical evidence in British primary schools. Nevertheless, a collaborative report by Waite et al. [21] considered how OAA could be implemented within a school setting and findings showed the schools most likely to facilitate OAA displayed characteristics such as strong leadership, open mindedness and the ability to create a positive staff culture [18,49]. Christie et al. [48] found teachers’ understanding of OAA varied, with many lacking awareness of the associated benefits, which was speculatively seen as a constraining factor.

Despite evidence clearly suggesting senior school staff play a significant role in advocating and supporting OAA implementation, it is concerning that Ofsted [18] highlighted “some schools remain unconvinced of the benefits (of OAA) when weighed against the many barriers” (p. 14). An Ofsted report [46] evaluating the impact of learning outside the classroom stated head teachers commonly reported staff, governors and parents had expressed concern that “time spent outside the classroom might reduce academic attainment” (p. 21). Further, Ofsted explained that some schools viewed OAA as an ‘extra’ or ‘special’ treat, confined to the limits of yearly off-site trips. School stakeholders often need reassurance of the integrity of OAA, and a strong rationale before incorporating this into curriculums [31]. Remington and Legge [43], however, also observed that, since outdoor centres often assume responsibility for school OAA delivery, it may be tangential to curricula with teachers becoming distanced from its associated educative potential.

An effective way to support schools with OAA delivery would be using funding to influence staff perspectives through professional development training [48] Thorburn and Allison [40] suggested sharing the growing evidence base and research credentials of OAA may help to convince others of its fidelity. Many scholars feel it necessary to challenge traditional perceptions of OAA within education and convey its numerous evidence-based benefits to all stakeholders involved [6,17,31,55]. Accordingly, Remington and Legge [43] found a strong enabling factor to be teachers’ awareness of the social benefits of OAA. Purposive support and development of teachers is an important theme many scholars have considered [6,7,17,31,40,55] and Christie et al. suggested OAA is gaining increasing recognition in schools [48].

## 3. Methodology

The methodology adopted a cross-sectional design to capture rich contextualised data from six primary schools and senior teachers across a large Northern city in England. Since the senior leadership teams of schools have been indicated as pivotal in shaping organisational practice and cultures [31], qualitative findings here specifically relate to educators interpretations of the OAA strand of the NC, understanding of OAA as a concept within an educational context, and sense of value placed on OAA within curriculum time. As this study was positioned in a social context with objectives largely based on socially constructed phenomena it incorporated an interpretivist paradigm to consider interpretations, personal emotions and values [20,59,60,61,62,63].

### 3.1. Participant Information

All participants were male and worked within senior leadership teams of primary schools holding responsibilities of overseeing and implementing OAA within curriculum time. Their teaching experience ranged from two to seventeen years, summarized below in Table 1. Considering some participants only had several years of teaching experience despite working within senior management teams, this potentially highlighted a lack of female or equivalent expertise and confidence in relation to PE and OAA within these primary schools.

### 3.2. Recruitment

Convenience sampling was used where participant inclusion criteria comprised of primary school teachers working within senior leadership teams overseeing and implementing OAA within curriculum time. A local school sport partnership (SSPa) organisation acted as a ‘gatekeeper’ for recruitment of senior teachers. Research information outlining thorough details of the study, participant expectations, the right to withdraw, and details of confidentiality measures were sent via email to a range of appropriate teachers selected by the gatekeeper. The researcher also attended a senior teacher conference hosted by the SSPa to invite participants to take part in a more personable manner. Informed consent was agreed and recorded prior to collecting data from all participants within interviews. In line with ethical approval granted via a University Ethics Committee and BERA guidelines [64], confidentiality and anonymity were offered to teachers and schools by the use of pseudonyms and stringent data protection etiquette.

### 3.3. Interviews

Semi-structured interviews were used to discuss real world topics and gain rich data [15,19,20,25,40,47,55,62]. An interview schedule was used throughout with prompts to elicit deeper responses (see Table 2), whilst also allowing conversation digression and unanticipated information to emerge [59,60,63]. Interview questions were informed by Curtner-Smith’s [50] early research on Occupational Socialization Theory, which considered influential factors on teachers’ interpretations of the NC. These original questions were adapted to fit the context and objectives of this study [61]. To enhance convenience for participants, interviews took place within schools over four weeks spanning January and February. Interview locations were private from children, including individual and shared offices, staff rooms, and vacant classrooms. Discussions lasted between 25 and 45 min and were audio recorded throughout to enable participants’ body language to be observed and any misunderstandings clarified. The semi-structured interview schedule used is presented below in Table 2.

### 3.4. Data Analysis

Qualitative data was analysed using a multi-stage thematic coding process to examine commonality, difference, and relationships [59,62,65,66]. First, interview recordings were transcribed verbatim before being thoroughly re-read to prompt familiarity with the data. Initial codes intended to encapsulate the main point being made were assigned to pertinent aspects of the transcripts in relation to the research objectives [66], with any irrelevant data being discarded from further analysis procedures [59,62]. These codes were successively processed deductively through alignment with the studies objectives to create overarching thematic categories. Inductive analysis was then used to capture the nuances within this data, with similar codes being grouped together to form subcategories [59,62]. Proposed themes and sub-categories were reviewed and refined to ensure findings accurately represented the dataset and clear distinctions existed [65]. This deductive–inductive approach allowed the macro focus of the original research objectives to be retained whilst also considering subsequent emergent findings [66]. To enhance the credibility of this process, colleague checks were performed throughout the analysis to substantiate any decisions made [67]. Similarly, the findings were also forwarded to all six participants to check the researcher’s interpretations were correct [62]. In keeping with confidentiality agreements, anonymised interview extracts were selected and used within the findings section to illustrate pertinent points made by participants [62].

## 4. Findings

This study aimed to investigate senior teachers’ interpretations, conceptual understanding and the value placed on primary education OAA. The findings integrate qualitative data from six senior teachers specifically related to the aim of this study.

### 4.1. Teachers Perceptions of OAA within the Curriculum

Senior teachers indicated the current NCPE was considerably slimmer and less prescriptive than earlier editions, with minimal inclusion of OAA. Five teachers expressed this allowed greater autonomy, yet another mentioned it suggested a lack of Governmental value. ”It’s gone from a curriculum that was pages and pages and very prescriptive … to schools having the autonomy that fits in with their school” (Bob). This lack of policy clarity was said to be replicated within OAA discourse since example activities are no longer provided within or alongside the curriculum forcing teachers to interpret the limited information provided. ”There’s not an awful lot to do with OAA on the curriculum is there … and it’s not tied down to particular events” (Dave). Three teachers mentioned this situation may be open to abuse by schools perceiving NCPE requirements as easy to fulfil, potentially creating a tokenistic approach. Equally, the majority of participants mentioned its sparseness and ambiguity allowed schools flexibility and was seen as a positive feature, highlighted below.

“There is not much there in terms of what we are supposed to be doing, but I think there is enough to take that and tailor it to offer something we want” (Bob).

Similarly, two teachers expressed the scant curriculum detail regarding OAA could pose a problem for less experienced generalist teachers and lacked ambition for more confident schools with well-established PE programmes. Consequently, the NCPE seemed to neither support the lower end of the spectrum regarding OAA confidence nor those schools aspiring to extend existing provisions shown here.

“Because it is so vague and doesn’t actually give any strategies of how to do it, your underqualified teachers, or your people that don’t have knowledge of OAA … don’t know how to do it” (Jerry).

In relation to teachers’ conceptual understanding of OAA, this was interpreted in a number of different ways. Two staff perceived OAA included ‘hard’ skills-based activities such as water sports and high ropes, while all participants suggested it incorporated lower level ‘softer’ activities focused on personal and social development. ”It’s things like hiking, climbing, canoeing, caving, and mountain biking” (Kevin) and ”it’s about building up their ability to work as a team” (Dave). Two senior teachers highlighted that these lower level interpersonal activities were markedly different to OAA typically delivered by schools in the past.

“I think historically it’s probably been OAA is done through residentials. There is a common misconception that OAA has to happen in mountainous areas, or on the lake or something, and it doesn’t really” (Bob).

While teachers did not always appear fully confidant regarding the conceptual characteristics of OAA, most seemed aware of the contrasting environment and pedagogical approach from typical classroom teaching illustrated here.

“That’s what OAA is about … an adventure … the chance to go and explore without me prescribing it … you give a little bit of structure … then you say, go on, off you go” (Jerry).

### 4.2. Values of OAA

This section outlines the sense of value senior teachers place on OAA within curriculum time and its perceived benefits, before considering several socio-cultural factors said to influence the status of OAA within primary education.

#### 4.2.1. School Based

All senior teachers appeared to highly value OAA and provided a strong rationale for its inclusion within the NC by identifying numerous benefits. These included developing pupils’ self-awareness, confidence, interpersonal skills and resilience, as articulated here: “I think OAA is massively valuable in terms of the teamwork, the friendships, and the relationships it can help to build” (Stuart). Teachers expressed that OAA developed life skills not explicitly taught within traditional education such as managing risk and becoming more independent. Likewise, this holistic form of education was also suggested to facilitate a great sense of achievement amongst students who may not typically experience this within academic spheres, shown here.

“Skills we take for granted a little bit because we don’t specifically teach them, like team working, being able to discuss a problem, being able to come up with ideas, and I think OAA is good for those sorts of things” (Bob).

Similarly, since several schools featured disadvantaged catchment areas in terms of socio-economic status, five teachers highlighted the importance of OAA in offering exploration and new experiences to students not readily available through their network of family and friends, as explained here: “There’s not a great deal students see other than brick walls … without school putting those OAA opportunities in place, most children wouldn’t experience it at all” (John).

Interestingly, three teachers also mentioned attempting to capitalise on the learning and development generated via OAA by transferring pertinent principles back into classrooms, as illustrated here:

“I firmly believe the OAA strand really builds resilience … if they have pushed themselves … we would talk about transferring that to when things are tough in literacy and you’re not quite getting it” (Dave).

These teachers clearly espoused a strong regard for OAA, with all providing significant advocacy within their respective organisations and attempting to convince other school stakeholders of its value and educative potential.

“You can probably tell I am a big fan of OAA … so I think whenever I have got a say in it, there will be a lot built in around it” (John).

#### 4.2.2. Socio-Cultural

Four teachers recognised their positive perceptions of OAA were not reflected amongst other educational professionals and suggested factors which may contribute to this situation, as expressed here: ”I would say from my experience that many schools place very little importance on OAA … it is neglected in the vast majority of primary and secondary schools” (Jerry). One example included the weight of accountability pressure schools face in relation to English and maths resulting in contrasting subject priorities. “Primary schools are dictated by maths and English results so that’s always going to be the main focus” (Stuart). Similarly, one teacher expressed that Ofsted places little value on both PE and OAA, which he found surprising when considering societal concerns such as childhood obesity and mental health. More recognition of the subject was said to be needed at governmental level before any change seemed likely, emotively explained here.

“It’s (OAA) undervalued because your school isn’t judged on it. You will be lucky if Ofsted even come out and check your PE lesson, let alone if you are doing OAA. It’s top down. If the government don’t place enough importance on it, then Ofsted don’t, and if Ofsted don’t … then your head teacher won’t … then you as a classroom teacher won’t do it because you will get criticised and asked why you are not in a classroom” (Jerry).

Having presented findings concerning teachers’ perceptions of OAA and how it is valued, the following discussion considers the data against other findings and its importance to OAA delivery in primary schools.

## 5. Discussion

### 5.1. Tensions of Professional Decision-Making

Since OAA coverage in the NCPE was highlighted as brief and ambiguous, this raised several concerns amongst senior teachers regarding professional decision-making, as illustrated here by Dave: ”There’s not an awful lot to do with OAA on the curriculum … and it’s not tied down to particular events”. As in research by MacLean et al. [53], some teachers subscribed to the enhanced autonomy offered by this slim policy document as lessons could be tailored to the needs of pupils and schools [53], as shown here by Bob: “I think there is enough to take that and tailor it to offer something we want”. This ideology should permit broad cross-curricular links to be made within OAA themed lessons. Equally, it is entirely possible this flexibility could be vulnerable to exploitation by some schools and potentially create a tokenistic approach towards OAA delivery as Flintoff indicated [56]. Other senior teachers suggested interpreting limited curricular information posed a problem for generalist or inexperienced teachers, as shown here by Jerry: “Because it is so vague … your underqualified teachers don’t know how to do it”. This argument aligned with findings by MacLean et al. [53] where teachers sought further clarification from senior management regarding policy interpretations due to unclear intentions and considerable flexibility [53]. Clearly, it seems policies can offer teachers too much freedom in unfamiliar areas, especially when not accompanied with formative feedback or support [53]. Considering many generalist teachers lack adequate subject and pedagogical knowledge in relation to OAA this raises several concerns [14,21,24,40,48,49,50,51,52,54]. Findings by Dyment et al. [55] substantiated this point as Australian teachers expressed anxiety due to curriculum documents lacking clarity and being too open to interpretation with no clear examples of OAA to inform delivery. Consequently, teachers were forced to interpret meaning by drawing on their personal experiences, something which Curtner-Smith [50] found resulted in considerably varied practice. Dyment et al. [55] concluded any curriculum changes should be supported by effective teaching resources, something not readily available for OAA in England at present. This situation ultimately equates to a lack of support for teachers and endorses the assertion of Sutherland and Legge [14] that educators require more thorough OAA preparation within initial teacher education programmes or professional development courses [40]. This lack of formal training [14,40] draws attention to the argument of participants that the NCPE lacks ambition for more confident schools aspiring to extend existing OAA provisions, as shown here by Kevin: “it’s not pushing people to have a very imaginative or exciting curriculum based on that one statement”. Many of these schools had a healthy culture of OAA delivery, yet seemingly also welcomed additional insight of new ideas or suggestions. This highlights the rationale for providing a range of OAA themed activities within or alongside the NCPE, ironically as was the case with the earlier policy document.

### 5.2. Teachers’ Conceptual Understanding of OAA

Senior teachers’ conceptual interpretations of OAA varied and encompassed both ‘hard’ skill-based activities and ‘soft’ activities focused on personal and social development. This finding aligns with existing literature [14,25,28] and highlights how some teachers subscribe to more traditional notions of OAA which involve skills focused outdoor pursuits incorporating elements of risk and challenge, as shown here by Kevin: “we talk to the children about managing the risks”. Since many activities of this type would be challenging for schools to provide onsite, this reflects a common misconception often associated with OAA that professional organisations facilitate delivery at purpose built outdoor centres [14,15,25,28,35]. Conversely, the ‘lower level’ component mentioned by participants is congruent with the Anglo-Saxon definition of OAA offered by Fagerstam [15], which referred to adventure experiences incorporating aspects of team building and leadership development. It was clear teachers were not always fully aware of OAA’s conceptual characteristics, yet understood it involved a contrasting setting and pedagogical approach to typical classroom teaching. This reflects regular references in literature of primary education teachers lacking clear conceptual understanding of OAA [14,21,24,28,40,48,49,50,51,52,54]. Nevertheless, participants seemed to grasp various overarching ideological principles of OAA and acknowledged this involved student-centred pedagogy with holistic multi-sensory experiences to promote exploration of outdoor phenomena with genuine consequences [7,15,17,23,68]. This general understanding amongst teachers is positive since literature indicates OAA can add context and depth to curricular with school experiences becoming more enjoyable, vivid, meaningful, and memorable by increasing students’ motivation and curiosity [18,22,46,68]. Similarly, the diverse range of perceptions regarding OAA potentially illuminates a shift in understanding amongst primary education teachers, as illustrated here by Kevin, who mentioned that team problem solving “is perhaps a bit different to what we did twenty years ago”. Thorburn and Allison [40] suggested diverse views like this can actually hinder the progress and implementation of OAA within education, although did not elaborate exactly how. Nevertheless, this situation highlights that OAA is a socially constructed subject [14,56] and influenced by various socio-cultural forces. Moving away from traditional notions of OAA centred around skill acquisition and risk align with a new vision posited by Dyment et al. [55] as more suitable for twenty-first century inclusion in curricular. This school-based ideology advocates cross-curricular learning in natural environments. This paradigm shift reflects the Scandinavian approach used for generations [15] and foregrounds the recent interest, remodeling and commercialisation of this approach marketed as ‘Forest Schools’ in England.

### 5.3. Value of OAA within Primary Education

All senior teachers highly valued OAA and provided a strong rationale for its place within the NC by identifying numerous associated benefits. These included aspects of personal and social development, gaining life skills not explicitly taught within traditional education, facilitating a sense of achievement amongst students who may not typically experience this within academic spheres, and offering opportunities that families may not be able to, summarised here by Stuart: ”I think OAA is massively valuable in terms of the teamwork, friendships, and relationships it can help to build”. This finding is congruent with the wealth of literature stating the ability of OAA to elicit personal and social development, as recognised here by teachers [16,18,19]. Slade et al. [20] highlighted collaborative learning can enhance interpersonal skills through greater social interaction during practical tasks. Similarly, Moreri [17] and Atencio et al. [25] suggest OAA is a good platform to disconnect young people from their habitual urban environments and assist in the development of more resilient, rugged, and self-sufficient citizens better able to function within society. This perspective mirrors aspects of the initial motivation to incorporate OAA into mainstream British education back in the 1970s [10]. These senior teacher perspectives also align with scholars such as Adams et al. [7] and Robinson [33] who suggest education is more than the acquisition of knowledge and has a moral obligation to educate children holistically for uncertain futures. This finding also substantiates Pether’s point [31] that OAA is no longer the domain of innovative schools but part of mainstream approaches to prepare young people for the workplace. Participants seemed aware of the unique potential of OAA to develop vital skills such as increased independence, teamwork, leadership and risk management [15,17,23]. Equally they also acknowledged the role of schools in offering inclusive provision to all pupils, as shown here by John: “without school putting those OAA opportunities in place, most children wouldn’t experience it”, which is important since Scrutton [19] stated children who may benefit most from OAA were often those least likely to afford it. This point aligns with Cook’s [10] previous assertion that OAA can unlock opportunities typically inaccessible to some socio-economic demographics. Moreover, Fagerstam [15] stated novel experiences can challenge typical classroom hierarchies of high and low achieving students by creating a new platform for pupils to show a different side to themselves and other abilities. Waite [23] found students often mentioned overcoming challenges during OAA led to a great sense of accomplishment, alluded to here by Bob: “you see children … really pushing themselves and … achieve something they thought they wouldn’t be able to”. Moreri [17] and Waite [23] both indicated that children often value freedom to explore natural environments finding it fun and exciting. Several scholars propose the dynamic OAA environment is capable of producing unanticipated learning opportunities that enhance student motivation, concentration and curiosity when compared to the classroom [15,20,28]. Similarly, the exploration, autonomy and creativity afforded by OAA have been shown to develop skills that can assist students with future life transitions [1,23,27,68]. Literature also suggests positive early OAA experiences can influence attitudes towards adventure, strenuous exercise, and the natural world, all vital in developing healthy lifelong habits [9,20,25,26].

Some teachers attempted to capitalise on the development achieved via OAA by transferring pertinent principles back into classrooms, as highlighted by Dave: “we would talk about transferring resilience to when things are tough in literacy and you’re not quite getting it”. The notion that participating in OAA can enhance academic attainment is becoming more prominent within literature [19], however, leveraging these benefits for school-based purposes is not often considered from a teacher’s perspective. One study by Atencio et al. [25] found many teachers felt learning from OAA did indeed transfer back into school classrooms, highlighting its importance as a future consideration. Despite admirable efforts by educators in this study, they were unable to offer robust insight into the effectiveness of this process. Nevertheless, this point is encouraging since the literature often indicates explicit links between OAA and classroom contexts are not attempted [14,35,46].

Considering the aggregation of positive factors associated with OAA offered by senior teachers, it was perhaps unsurprising they provided significant advocacy for this within their respective schools, as shown here by John: “whenever I have got a say in it, there will be a lot built in around OAA”. This finding is congruent with numerous references in literature regarding senior teachers being the main driving force within schools and their enthusiasm cascading down to inspire others [21,31,48,49]. This support was sometimes a collaborative effort by several staff, but also individuals, reflecting Pether’s [31] point of senior leadership being instrumental in successful development of OAA cultures. Similarly, a finding by Waite et al. [21] was also pertinent, highlighting the schools most likely to implement OAA were those with a positive staff culture towards the subject.

Participants also suggested several socio-political factors which influenced the value of OAA within schools. For example, other teachers and organisations were suggested to not share their positive vision for OAA, the weight of accountability pressure in English and maths often resulted in OAA being neglected, and a lack of focus by Ofsted was cited to compound this situation, alluded to here by Jerry: “from my experience OAA is neglected in the vast majority of primary and secondary schools”. Despite OAA now being a compulsory part of the curriculum, this finding aligns with literature [18,46] which indicates many school stakeholders are unconvinced of OAA’s place within schools as it may detract from learning occurring within classrooms. Components of Pether’s study [31] reflect this as school stakeholders required reassurance of OAA’s integrity and rationale before incorporating this into curricular. Several scholars attributed a lack of value regarding OAA to deficient understanding of the subject’s purpose and educational credentials [17,20]. Curtner-Smith suggested [50] if teachers’ early life experiences lacked the affordance of OAA then it seemed likely their interpretations of PE may favour more familiar and traditional sports. Dyment et al. [55] indicated this concern extends beyond a British context, with Cosgriff [28] explaining OAA is experiencing similar challenges in Australasia. Despite such difficulties, a strategy touted as powerful enough to convince sceptics involves sharing OAAs compelling rationale and growing empirical evidence base alongside its impact on both pupils and teachers [40,46]. Such an approach, however, would involve pushing against the performative and comparative agenda within education that champions attainment across core subjects such as English and maths. The seminal paper by Ball [41] indicated success in education has recently been measured by these indicators and resulted in significant pressures of accountability, thus prompting schools to strive for this narrow measure of success at all costs. Cosgriff [28] mentioned that the introduction of National priorities requiring teachers to report on students’ literacy and maths progress can significantly impact other curricular components such as OAA. Cosgriff stated this leads to higher staff workload and a narrower curriculum, which Spielman [44] asserted is sadly the case within primary education in England. Dyment et al. [55] argued this narrow focus reinforces decontextualized classroom learning and the marginalisation of subjects historically considered less academic. Ofsted have recently recognised ramifications stemming from the current performance-based education system and attempted to counterbalance these within a new school inspection framework. This will soon require organisations to explicitly demonstrate a broad and balanced curriculum, with attention directed towards subject leaders regarding PE [69]. Once implemented this legislation has potential to mitigate these concerns; however, at present, it has received only sceptical support from professionals.

## 6. Conclusions

The purpose of the study was to investigate how senior teachers within primary education in England interpreted the OAA strand of the NC, understood OAA as a concept within an educational context, and valued this approach within curriculum time. The methodology replicated other similar studies conducted around the world [15,19,20,25,40,47,55].

The recent Conservative Government in England have dramatically reduced content of the NC for foundation subjects such as PE to encourage professional decision-making. This shift has resulted in policy unable to meet the needs of teachers at both ends of the confidence spectrum regarding OAA delivery. These findings indicate contemporary legislation has regressed in terms of supporting teachers in this area. Governmental aspirations for greater teacher autonomy seem a commendable intention. However, when this involves a curricular area such as OAA associated with generalist teachers lacking expertise and confidence, its outcome is problematic. Findings of this study suggest teachers clearly require more support interpreting the vague and ambiguous NC information available for OAA. The lack of conceptual clarity regarding OAA amongst senior staff, coupled with generalist teachers struggling to interpret the sparse curriculum and its lack of value amongst other educators illuminates the salience of professional development training.

Recent socio-political shifts within education may offer some legitimacy to any sceptical school stakeholders regarding the value of OAA. These include acknowledgement by Ofsted that primary education curricula are too narrow and the implementation of a new school inspection framework to evaluate whether schools are providing a broad and balanced curriculum.

This study draws attention to several challenges within primary education for the delivery of OAA and outlines broad strategies in response. Its intentions are to ensure teachers feel better informed and supported in relation to OAA and encourage more schools to consider and engage with this holistic form of education. As minimal research has focused on factors which influence OAA within primary education in England since the recent curriculum change and introduction of the SSPr, this study offers new empirical findings to an underdeveloped literature base [19,25,28,55]. Despite contextualised findings being unable to represent the wider population, pertinent points can be considered and potentially transferred to other similar settings [62,67]. This can inform future work of policy makers, practitioners, and scholars within the UK and further afield.

### 6.1. Limitations

Study limitations have been noted. For example, participants were a non-representative sample, since all schools featured enthusiastic staff who championed OAA delivery within their respective organisation. The invitation to participate in this study appealed to senior teachers already interested in the outdoors. The sample included male senior teachers, which may be seen as unrepresentative of a predominantly female workforce within primary education. Senior teachers in primary schools hold considerable influence over school practices, yet the scope and size of this study means the research is a snapshot of OAA delivery in the North of England. While transferability to other similar educational settings may be considered, a greater evidence base is required in order to more fully understand OAA and its place in primary education in England.

### 6.2. Recommendations

NC documentation has to provide a more robust OAA framework to instil confidence amongst primary education teachers. It needs to emphasise a range of suitable activities and associated pedagogical approaches, beginning with the use of immediate school environments to help dispel the myth that OAA is just about “hiking, climbing, caving and mountain biking” (Kevin). Such an approach was adopted in the past before the inception of the 2013 NC with good effect. Creating guidance to supplement the NC would require consultation from the many OAA organisations across the UK to ensure support is informed by both research and practice. Similarly, the SSPr could also be used for professional development purposes and result in schools becoming more self-sufficient with OAA and enhance the longitudinal impact of this potentially ephemeral revenue stream. This approach could be further supported by introducing senior peripatetic teachers with OAA experience and interest who could coordinate delivery across several schools concurrently. Such considerations require acknowledgment within initial teacher education, with fundamental changes required. The compulsory element of OAA in primary schools, which often includes some form of residential experience, should be reflected within teacher development programmes. This could be purposefully integrated by explicitly outlining OAA’s educative potential, conceptual characteristics, curricular requirements, pedagogical principles and demystifying appropriate activities. While ambitious, programmes could also consider providing short introductory OAA themed residentials organised by pre-service teachers early in the programme as an introduction to OAA and means of developing course culture and strong bonds amongst peers. Subsequent cross-curricular day visits could also be organised to showcase the strengths of OAA as a legitimate approach to learning. Schools should also be encouraged to support pre-service teaches engaging with OAA opportunities available within their professional teaching placements.

More empirical research of OAA in primary education, specifically considering women who represent the majority of teachers and their experiences is recommended. Similarly, more could be done to capture the exemplary practice and worth of OAA in primary schools to create a more positive culture within education.

## Figures and Tables

**Table 1:** Summary of participant characteristics.

Pseudonym	Role	Experience (Years)
Kevin	Head Teacher	17
Stuart	Assistant Head Teacher	12
Jerry	PE Coordinator	6
John	PE Coordinator	5
Bob	PE Coordinator	3
Dave	PE Coordinator	2

**Table 2:** Interview schedule.

Question	Prompts
How long have you worked as a teacher and senior teacher?	What about at this school?
What do you interpret the OAA strand of the PE curriculum to mean?	Could you talk a little about the clarity of the wording used in the NC?
–	Is there any additional guidance provided alongside the NC in relation to OAA?
–	What do you think about the policy in relation to generalist teachers?
What sense of value do you place on OAA within curriculum time?	How does this compare with the perspectives of other school stakeholders?
–	Are you aware of any of the benefits of OAA?
Is there anything else you would like to add on this topic?	–

## Glossary of Terms

NC	National Curriculum
PE	Physical Education
NCPE	Physical Education National Curriculum
KS	Key Stage
OE	Outdoor Education
OAA	Outdoor and Adventurous Activities
SSPa	School Sports Partnership
SSPr	School Sports Premium

## References

[1] The Outward-Bound Trust (2014). Social Impact Report. https://www.outwardbound.org.uk/media/546349/social-impact-report-2014.pdf.

[2] Kings College London Understanding the Diverse Benefits of Learning in Natural Environments. http://publications.naturalengland.org.uk/publication/4524600415223808.

[3] Sharma-Brymer V., Bland D. (2016). Bringing Nature to Schools to Promote Children’s Physical Activity. Sports Med..

[4] H.M. Government A Green Future: Out 25 Year Plan to Improve the Environment. https://assets.publishing.service.gov.uk/government/uploads/system/uploads/attachment_data/file/693158/25-year-environment-plan.pdf.

[5] Beames S., Brown M. (2016). Adventurous Learning A Pedagogy for a Changing World.

[6] Wattchow B., Brown M. (2001). A Pedagogy of Place—Outdoor Education for a Changing World.

[7] Adams K., Monahan J., Wills R. (2015). Losing the whole child? A national survey of primary education training provision for spiritual, moral, social and cultural development. Eur. J. Teach. Educ..

[8] Brown M., Kirk D., Macdonald D., O’Sullivan M. (2006). Adventure Education and Physical Education. The Handbook of Physical Education.

[9] Clough P., Mackenzie S.H., Mallabon L., Brymer E. (2016). Adventurous Physical Activity Environments: A Mainstream Intervention for Mental Health. Sports Med..

[10] Cook L. (2001). Differential Social and Political Influences on Girls and Boys through Education Out of Doors in the United Kingdom. J. Adventure Educ. Outdoor Learn..

[11] Cooper S., Jeffs T., Ord J. (2018). ‘Living Together’ Making the most of residential experience in outdoor and adventure education. Rethinking Outdoor, Experiential and Informal Education.

[12] Hunt J.S. (1990). Ethical Issues in Experiential Education.

[13] Legge M.F., Smith W. (2014). Teacher Education and Experiential Learning: A Visual Ethnography. Aust. J. Teach. Educ..

[14] Sutherland S., Legge M. (2016). The Possibilities of “Doing” Outdoor and/or Adventure Education in Physical Education/Teacher Education. J. Teach. Phys. Educ..

[15] Fagerstam E. (2014). High School Teachers Experience of the Educational Potential of Outdoor Learning. J. Adventure Educ. Outdoor Learn..

[16] McKenzie M. (2003). Beyond ‘The Outward-Bound Process’: Rethinking student learning. J. Exp. Educ..

[17] Moreri A.B. (2011). Challenges in the Delivery of Adventure Education: The Case of Botswana Junior Secondary Schools. S. Afr. J. Res. Sport Phys. Educ. Recreat..

[18] Ofsted Outdoor Education Aspects of Good Practice. http://www.docs.hss.ed.ac.uk/education/outdoored/ofsted_oe_report_2004.pdf.

[19] Scrutton R.A. (2015). Outdoor Adventure Education for Children in Scotland: Quantifying the Benefits. J. Adventure Educ. Outdoor Learn..

[20] Slade M., Lowery C., Bland K. (2013). Evaluating the Impact of Forest Schools: A Collaboration Between a University and a Primary School. Br. J. Learn. Support.

[21] Waite S., Passy R., Gilchrist M., Hunt A., Blackwell I. Natural Connections Demonstration Project, 2012–2016: Final Report. http://publications.naturalengland.org.uk/publication/6636651036540928.

[22] Learning and Teaching Scotland (2010). Curriculum for Excellence Through Outdoor Learning. https://education.gov.scot/Documents/cfe-through-outdoor-learning.pdf.

[23] Waite S. (2007). Memories are Made of This: Some Reflections on Outdoor Learning and Recall. J. Educ..

[24] Kings College London Beyond Barriers to Learning Outside the Classroom in Natural Environments. http://publications.naturalengland.org.uk/publication/4524600415223808.

[25] Atencio M., Ho S., Ching C.T., Michelle Tan Y.S. (2015). ‘The Strawberry Generation… They are too Pampered’: Pre-service Physical Education Teachers’ Perspectives on Outdoor Education in Singapore. Eur. Phys. Educ. Rev..

[26] Kirk D. (2005). Physical education, youth sport and lifelong participation: The importance of early learning experiences. Eur. Phys. Educ. Rev..

[27] Allan J., McKenna J., Buckland H., Bell R. (2014). Getting the right fit: Tailoring Outdoor Adventure Residential Experiences for the Transition of School Children. Phys. Educ. Matters.

[28] Cosgriff M. (2016). The Reconceptualisation of Outdoor Education in the Primary School Classroom in Aotearoa New Zealand: How might we do it?. J. Educ..

[29] Department for Education National Curriculum in England, Physical Education Programmes of Study: Key Stages 1 and 2. https://assets.publishing.service.gov.uk/government/uploads/system/uploads/attachment_data/file/239040/PRIMARY_national_curriculum_-_Physical_education.pdf.

[30] Martin P., McCullagh J. (2011). Physical Education and Outdoor Education: Complementary but discrete disciplines. Asia-Pac. J. Health Sport Phys. Educ..

[31] Pether T. Leadership for Embedding Outdoor Learning Within the Primary Curriculum. http://www.lotc.org.uk/wp-content/uploads/2012/04/Leadership-for-embedding-outdoor-learning-within-the-primary-curriculum.pdf.

[32] Potter T.G., Dyment J.E. (2016). Is Outdoor Education a Discipline? Insights, Gaps, and Future Directions. J. Adventure Educ. Outdoor Learn..

[33] Robinson K. Do Schools Kill Creativity?. https://www.ted.com/talks/ken_robinson_says_schools_kill_creativity?language=en.

[34] Sutherland S., Stuhr P.T. (2014). Reactions to implementing adventure-based learning in physical education. Sport Educ. Soc..

[35] Thorburn M., Allison P. (2010). Are we ready to go outdoors now? The prospects for outdoor education during a period of curriculum renewal in Scotland. Curric. J..

[36] Zmundy M.H., Curtner-Smith M.D., Steffen J. (2009). Influence of occupational socialization on the practices and perspectives of two inexperienced adventure educators. J. Adventure Educ. Outdoor Learn..

[37] Department for Education Initial Teacher Training Criteria and Supporting Advice. https://www.gov.uk/government/publications/initial-teacher-training-criteria/initial-teacher-training-itt-criteria-and-supporting-advice.

[38] H.M. Treasury Budget 2016. https://assets.publishing.service.gov.uk/government/uploads/system/uploads/attachment_data/file/508193/HMT_Budget_2016_Web_Accessible.pdf.

[39] Ofsted The PE and Sport Premium for Primary Schools. https://assets.publishing.service.gov.uk/government/uploads/system/uploads/attachment_data/file/379202/The_20PE_20and_20sport_20premium_20for_20primary_20schools.pdf.

[40] Thorburn M., Allison P. (2013). Analysing Attempts to Support Outdoor Learning in Scottish Schools. J. Curric. Stud..

[41] Ball S.J. (2010). The teacher’s soul and the terrors of performativity. J. Educ. Policy.

[42] Cramp A. (2008). Knowing me knowing you: Building valuable relationships outside the classroom. Int. J. Prim. Elem. Early Years Educ..

[43] Remington T., Legge M. (2017). Outdoor Education in Rural Primary Schools in New Zealand: A Narrative Enquiry. J. Adventure Educ. Outdoor Learn..

[44] Spielman A. Amanda Spielman’s speech at Ark’s Teach 2017 Conference. https://www.gov.uk/government/speeches/amanda-spielmans-speech-at-arks-teach-2017-conference.

[45] Brown M. (2008). Comfort Zone: Model or Metaphor?. Aust. J. Outdoor Environ. Educ..

[46] Ofsted Learning Outside the Classroom, How Far Should You Go?. https://www.lotc.org.uk/wp-content/uploads/2010/12/Ofsted-Report-Oct-2008.pdf.

[47] Thomas G. (2015). Signature Pedagogies in Outdoor Education. Asia-Pac. J. Health Sport Phys. Educ..

[48] Christie B., Beames S., Higgins P., Nicol R., Ross H. (2014). Outdoor Learning Provision in Scottish Schools. Scott. Educ. Rev..

[49] Ofsted Beyond 2012—Outstanding Physical Education for All. https://assets.publishing.service.gov.uk/government/uploads/system/uploads/attachment_data/file/413187/Beyond_2012_-_outstanding_physical_education_for_all.pdf.

[50] Curtner-Smith M.D. (1999). The More Things Change the More They Stay the Same: Factors Influencing Teachers Interpretations and Delivery of National Curriculum Physical Education. Sport Educ. Soc..

[51] Houlihan B., Green M. (2006). The changing status of school sport and physical education: Explaining policy change. Sport Educ. Soc..

[52] Whipp P.R., Hutton H., Grove R., Jackson B. (2017). Outsourcing Physical Education in Primary Schools: Evaluating the impact of externally provided programmes on generalist teachers. Asia-Pac. J. Health Sport Phys. Educ..

[53] MacLean J., Mulholland R., Gray S., Horrell A. (2015). Enabling curriculum change in physical education: The interplay between policy constructors and practitioners. Phys. Educ. Sport Pedagog..

[54] Carney C., Armstrong N. (1996). The Provision of Physical Education in Primary Initial Teacher Training Courses in England and Wales. Eur. Phys. Educ. Rev..

[55] Dyment J., Morse M., Shaw S., Smith H. (2014). Curriculum Development in Outdoor Education: Tasmanian Teachers Perspectives on the New Pre-Tertiary Outdoor Leadership Course. J. Adventure Educ. Outdoor Learn..

[56] Flintoff A., Hylton K. (2013). Sports Development Policy, Process and Practice.

[57] Penny D., Evans J., Green K., Hardman K. (2005). ‘Policy, power and politics in physical education’. Physical Education: Essential Issues.

[58] Penny D., Evans J. (1999). Politics, Policy and Practice in Physical Education.

[59] Gratton C., Jones I. (2010). Research Methods for Sport Studies.

[60] Greetham B. (2014). How to Write your Undergraduate Dissertation.

[61] Jones I., Brown L., Holloway I. (2013). Qualitative Research in Sport and Physical Activity.

[62] Sparkes A.C., Smith B. (2014). Qualitative Research Methods in Sport, Exercise and Health.

[63] Winstanley C. (2010). Writing a Dissertation for Dummies.

[64] British Educational Research Association Ethical Guidelines for Educational Research. https://www.bera.ac.uk/wp-content/uploads/2014/02/BERA-Ethical-Guidelines-2011.pdf?noredirect=1.

[65] Braun V., Clarke V. (2006). Using thematic analysis in psychology. Qual. Res. Psychol..

[66] Harding J. (2013). Qualitative Data Analysis from Start to Finish.

[67] Guba E., Lincon Y. (1989). Fourth Generation Evaluation.

[68] Hawman C. (2012). OAA Matters. Phys. Educ. Matters.

[69] Ofsted Chief Inspector sets out vision for new Education Inspection Framework. https://www.gov.uk/government/news/chief-inspector-sets-out-vision-for-new-education-inspection-framework.

